# Blood pressure change and hypertension incidence among Ghanaians living in rural Ghana, urban Ghana and The Netherlands: a prospective cohort study

**DOI:** 10.1016/j.eclinm.2025.103141

**Published:** 2025-03-05

**Authors:** Eva L. van der Linden, Marieke Hoevenaar-Blom, Erik Beune, Samuel Nkansah Darko, Sampson Twumasi Ankrah, Karlijn A.C. Meeks, Felix Chilunga, Charles Hayfron-Benjamin, Peter Henneman, Bert-Jan van den Born, Ellis Owusu Dabo, Charles Agyemang

**Affiliations:** a Department of Public and Occupational Health, Amsterdam UMC, University of Amsterdam, Amsterdam Public Health Research Institute, Amsterdam, The Netherlands; b Department of General Practice, Amsterdam UMC, University of Amsterdam, Amsterdam, The Netherlands; c Department of Molecular Medicine, Kwame Nkrumah University of Science and Technology (KNUST), Kumasi, Ghana; d Department of Statistics and Actuarial Science, Kwame Nkrumah University of Science and Technology (KNUST), Kumasi, Ghana; e Center for Research on Genomics and Global Health, National Human Genome Research Institute, National Institutes of Health, Bethesda, MD, USA; f Department of Physiology, University of Ghana Medical School, Accra, Ghana; g Department of Anesthesia and Critical Care, Korle Bu Teaching Hospital, Accra, Ghana; h Department of Human Genetics, Epigenetics, Reproduction & Development, Amsterdam UMC, University of Amsterdam, Amsterdam, The Netherlands; i Department of Vascular Medicine, Amsterdam UMC, University of Amsterdam, Amsterdam Cardiovascular Sciences, Amsterdam, The Netherlands; j School of Public Health, Kwame Nkrumah University of Science and Technology (KNUST), Kumasi, Ghana

**Keywords:** Hypertension, Blood pressure, Migrant, Ghana

## Abstract

**Background:**

Longitudinal data on blood pressure changes in sub-Saharan African populations are limited despite a high hypertension burden. This study analysed systolic blood pressure (SBP) change and hypertension incidence among people from Ghana living in rural Ghana, urban Ghana, people from Ghana living in The Netherlands and a Dutch European population living in Amsterdam, The Netherlands.

**Methods:**

The population-based Research on Obesity and Diabetes among African Migrants Prospective (RODAM-Pros) cohort study included adults aged ≥18 years at baseline (2012–2015) and follow-up (2019–2021) to study cardiovascular risk factors. At both timepoints, blood pressure (BP) was measured using a semiautomated device. Hypertension was defined as having a SBP ≥ 140 mmHg, diastolic BP ≥ 90 mmHg or the use of antihypertensive medication. We compared age-standardised SBP change and hypertension incidence between the geographical locations via linear and Poisson regression analyses, with adjustment for age, follow-up time, education, baseline BP, body mass index, renal function, and diabetes mellitus. The study protocol was approved by the respective ethics committees in Ghana and The Netherlands.

**Findings:**

Data from 632 people living in rural Ghana, 602 in urban Ghana, 861 Ghanaian, and 2038 Dutch people living in Amsterdam, were analysed (58.3% women, mean age at baseline 46.5 years, follow-up time 6·5 years). SBP increased most in women in rural Ghana (+9.5 mmHg, 95% confidence interval 7·3–11·7 mmHg), compared to +5·7 mmHg (3·6–7·7 mmHg) in urban Ghana, +2·2 mmHg (0·7–3·7 mmHg) in Ghanaian women in Amsterdam and −0·4 mmHg (−1·2 to 0·4 mmHg) in Dutch women. In men, SBP increased +5·5 mmHg (2·6–8·4 mmHg) in rural Ghana, +6·1 mmHg (2·8–9·5 mmHg) in urban Ghana, +2·1 mmHg (0·4–3·8 mmHg) in Ghanaian men in Amsterdam, and +0·3 mmHg (−0·5 to 1·1 mmHg) in Dutch men. Hypertension incidence ranged from 20·7% (95% confidence interval 14·3–29·2%) in men in rural Ghana to 34·2% (23·3–49·1%) in urban Ghana, vs. 27·9% (19·8–38·5%) in Ghanaian men in Amsterdam and 14·5% (11·8–17·6%) in Dutch men. Among women, incidence was 29·0% (23·1–35·9%) in rural Ghana, 27·6% (21·4–35·3%) in urban Ghana, 34·4% (26·0–45·4%) in Ghanaian women in Amsterdam, and 7·2% (5·6–9·2%) in Dutch women. Hypertension incidence rate ratios did not differ across populations, regardless of adjustment for covariates.

**Interpretation:**

SBP and hypertension increases were more pronounced in rural and urban Ghana than among migrants from Ghana in The Netherlands, suggesting that urbanisation of cardiovascular risk profile now extends to rural sub-Saharan Africa.

**Funding:**

10.13039/501100000781European Research Council (grant number 772244).

**Research in context**

**Evidence before this study**

While hypertension is highly prevalent among populations of West African origin, there are substantial differences in prevalence rates across settings, such as rural and urban settings, as well as for people who migrated from West African to high-income regions. We searched PubMed in September 2022 for articles describing longitudinal cohort studies performed in sub-Saharan Africa (SSA), and for studies including SSA migrants in high-income countries, using a combination of MeSH terms on “Blood Pressure”, “Hypertension”, “Africa South of the Sahara”, “Transients and Migrants”, and “Cohort Studies”. In the search results, we focussed on publications describing populations from West African or Ghana, if available. The search yielded studies describing historical trends in blood pressure and hypertension prevalence for different world regions, showing an increasing mean blood pressure and hypertension burden in SSA in the past decades. For example, a 5-year prospective study among Black South Africans aged >30 years reported a hypertension incidence of 24% amongst those with a BP of ≤120/80 mmHg at baseline. However, we found no population-based prospective cohort studies that followed people from West Africa living in diverse settings over time.

**Added value of this study**

This prospective cohort study, including Ghanaians living in rural and urban settings in Ghana and Ghanaians living in Europe, shows that increase in systolic blood pressure and in hypertension prevalence are more pronounced in urban and rural Ghana, compared to Ghanaians living in Amsterdam. In rural Ghana, hypertension prevalence increased from 26% to 34% in men and from 34% to 46% in women, over a period of 6·6 years follow-up. In men in urban Ghana, hypertension prevalence increased from 42% at baseline to 54% at follow-up, and from 38% at baseline to 48% at follow-up in women in urban Ghana. Our results may suggest that the “urbanisation” of lifestyle and cardiovascular risk profile has reached rural areas in Ghana.

**Implications of all the available evidence**

In 2013, the World Health Organization set the goal to reduce the prevalence of hypertension by 25% by the year 2025, compared to 2010, as part of the Global Non-Communicable Disease Action Plan 2013–2020. The observed high and increasing prevalence of hypertension in both rural and urban settings in Ghana calls for critical reassessment of the current organisation of hypertension preventative efforts. Future efforts should go beyond individual level lifestyle advice and address system level factors, such as neighbourhood urban design and food environment, contributing to disparities in hypertension burden. More longitudinal cohorts are needed in the SSA context, as well as multi-ethnic cohorts in Europe, to evaluate time trends and drivers of blood pressure change, hypertension and hypertension-related complications.

## Introduction

Hypertension is the major modifiable risk factor for cardiovascular diseases (CVD), contributing to 22·3% of the global CVD prevalence,^1^ and to 7·8% of the overall disease burden.^2^ The burden of hypertension is specifically high among migrants from sub-Saharan Africa (SSA) in Europe. They have higher mean levels of systolic and diastolic blood pressure (BP),^3^ and the prevalence of hypertension is up to 3·5 times higher in migrants from SSA compared to the European population without migration background.^4^ Additionally, in the SSA region, the prevalence of hypertension is increasing^5^ and expected to rise further if preventive measures are not put into place.^6^

Factors driving this high burden of hypertension among populations from SSA are still incompletely understood, and do not fully explain the difference in burden of hypertension between migrants from SSA and the European host population.^7^ Additionally, the factors influencing hypertension differ between migrants from SSA in Europe and non-migrants residing in rural and urban settings in SSA.^8^

Longitudinal studies describing BP changes, hypertension incidence and influencing factors will help to identify key drivers behind the high burden of hypertension among populations from SSA. However, such evidence is currently limited. By comparing change in BP and hypertension incidence between migrant populations from SSA residing in Europe and their non-migrating counterparts in SSA, the impact of migration and context on BP and hypertension can be studied, and results can inform healthcare policies and preventative measures in both settings. By comparing migrant populations from SSA to European host populations, ethnic differences in hypertension incidence can be examined, and this can inform efforts to mitigate inequalities in health.

Therefore, the aim of this study was to examine the change in BP over time and the incidence of hypertension among migrants from Ghana residing in Amsterdam, The Netherlands, compared to non-migrants residing in urban and rural Ghana, and the Dutch population without migration background. Furthermore, we assessed whether the differences between populations could be explained by conventional risk factors for hypertension. We hypothesised that the change in BP would be larger in Ghanaians in urban Ghana and Amsterdam than in rural Ghana, as the BP and hypertension prevalence at baseline showed a positive gradient from rural Ghana, through urban Ghana, to Amsterdam.^9^ Compared to the Dutch population without migration background, we expected to find a larger change in BP and higher hypertension incidence rates in Ghanaians in Amsterdam, as previous studies have shown large differences in hypertension and BP between these groups.^3^^,^^4^

## Methods

Details of the multicentre Research on Obesity and Diabetes among African Migrants Prospective (RODAM-Pros) cohort study design, recruitment and data collection have been published elsewhere^10^ and will be summarised here.

### Study population and study design

The population-based RODAM-Pros study included Ghanaians residing in rural and urban Ghana, and in Amsterdam, The Netherlands, and was designed to identify key changes in environmental exposures (e.g., socioeconomic status, lifestyle factors, psychosocial factors), biological factors and epigenetic modifications in the development of cardiovascular risk.^10^ The majority (83·5%) of the participants was of Akan ethnicity, the largest ethnic group in Ghana.^11^ All 5114 participants (1111 rural Ghana, 2183 urban Ghana, 2551 Ghanaians in Amsterdam) of the RODAM cross-sectional study that completed baseline data collection (1st July 2012 till 1st October 2015), were eligible for follow-up inclusion in the RODAM-Pros study (July 1st 2019 till November 1st 2021).

At baseline, participants in Ghana were selected from 15 villages and 2 cities, from a list of enumeration areas in the Ashanti region. Ghanaian participants in The Netherlands were randomly drawn from the Amsterdam municipal register, based on the country of birth of each citizen and that of their parents. Participants were referred to as “Ghanaian” if they were born in Ghana and their parents were born in Ghana; participants were referred to as “Dutch” if they were born in The Netherlands and their parents were born in The Netherlands. At follow-up, in rural Ghana, all study participants were contacted by home visits, and by phone calls in urban Ghana. In The Netherlands, all study participants were sent a written invitation and received phone calls. Additionally, a randomly selected subpopulation of Dutch participating in the Healthy Life in an Urban Setting (HELIUS) was included in the RODAM-Pros study. This was possible, as both studies used the same study methods.

The response rates at follow-up were 63% in rural Ghana, 44% in urban Ghana, 68% in Ghanaians in Amsterdam, and 93% in Dutch. 91% of the respondents in rural Ghana, 95% in urban Ghana, 53% of Ghanaians in Amsterdam, and 67% of Dutch completed the physical examination.

### Ethics

Ethical approval of the study protocols was obtained from the respective ethics committees in Ghana (School of Medical Sciences/Komfo Anokye Teaching Hospital Committee on Human Research, Publication & Ethical Review Board, reference CHRPE/AP/172/19), and Amsterdam (Institutional Review Board of the Academic Medical Centre, University of Amsterdam, reference NL32251.018.10). Participants gave informed consent to participate in the study before taking part.

### Measurements

Standardised protocols were used for data collection, to ensure comparability of the results between the different study populations and between baseline (2012–2015) and follow-up data collection (2019–2021). See Supplementary File S1 for detailed description of the measurements taken and definitions used.

In short, during a structured, in-person interview performed by trained ethnically matched research personnel, data including demographics (sex assigned at birth, age, length of stay in Europe), level of education, family history of hypertension, physical activity, smoking, daily energy, alcohol and sodium intake, psychosocial stress, and depressive symptoms were collected. Dutch participants completed an online digital Dutch version of the structured health questionnaire.

During physical examination, anthropometric measures (weight, height, waist-, and hip-circumference) and blood pressure measurements were taken at least twice, and the mean of the first two readings were used for analyses. Using a validated semiautomated device (WatchBP Home, Microlife AG Swiss Corporation, Widnau, Switzerland), BP was measured in a sitting position, after at least five minutes rest, using an appropriate cuff size on the left upper arm. Hypertension was defined as having a systolic BP (SBP) of ≥140 mmHg or a diastolic BP (DBP) of ≥90 mmHg or the use of antihypertensive medication as classified by Anatomical Therapeutic Chemical code.

Venous blood samples were collected after an overnight fast of at least ten hours, and fasting plasma glucose and plasma creatinine were measured, to assess diabetes mellitus status and estimated glomerular filtration rate (eGFR), respectively.

### Statistics

Baseline population characteristics were described using counts and percentages for categorical variables, mean and standard deviation for normally distributed (determined by visual inspection of the histograms), and median and interquartile range for non-normally distributed continuous variables. To compare baseline characteristics between the geographical locations we used one-way analysis of variance for normally distributed continuous variables, Kruskal–Wallis test for non-normally distributed continuous variables, and Chi-square test for categorical variables. Because of statistically significant interaction (p-value of interaction term <0·05) between site and sex and incident hypertension (outcome), all analyses were stratified by sex. No other interactions were tested.

Change in BP was adjusted for the use of BP-lowering medication by adding 10 mmHg to SBP, and 5 mmHg to DBP (at both time points) if the participant used BP-lowering medication.^12^ Age-standardisation was performed with the distribution of age at baseline of the total population, for men and women, to correct for age difference between geographical locations.

The age-standardised 6-years hypertension incidence proportion (cumulative incidence rate) was calculated for those without hypertension at baseline, using the direct method. Additionally, age-standardised incidence rates per 1000 person-years were calculated.

To compare the change in SBP between the geographical locations, linear regression was performed, with rural Ghana being the reference population, as we hypothesised this population to have the smallest change in SBP, and rural Ghana being conceptualised as the source location from which participants migrated to urban Ghana and subsequently to Europe. Three different models were fitted to assess the impact of sociodemographic and health factors on the association between geographical location and change in SBP. Model 1 included geographical location (predictor), age at baseline, follow-up time in years, and SBP at baseline. Model 2 additionally included level of education as a proxy for socioeconomic status. Model 3 additionally included baseline body mass index (BMI), estimated glomerular filtration rate (eGFR) and diabetes mellitus to adjust for conventional determinants of BP. The same models were fitted to compare change in SBP between Ghanaians in Amsterdam and Dutch, with the Dutch as the reference population.

Similarly, the incidence of hypertension was compared between the geographical locations, using Poisson regression in three models, with people in rural Ghana as the reference population. Model 1 was adjusted for age at baseline, follow-up time in years, SBP and DBP at baseline; model 2 was additionally adjusted for level of education; model 3 was additionally adjusted for BMI, eGFR and diabetes mellitus. Also, hypertension incidence was compared between Ghanaians in Amsterdam and Dutch, with the Dutch as the reference population, using the same models.

Several sensitivity analyses were performed. Firstly, the linear and Poisson regression analyses were performed in additional models to assess the effect of various determinants of BP and hypertension on the geographical comparison. Model 2b adjusted for the factors included in model 2 plus change in BMI between baseline and follow-up, as change in BMI is an important predictor for change in BP.^13^ Model 3b additionally adjusted model 3 for WHR. Model 4 adjusted for model 3 plus family history of hypertension to adjust for shared genetics and environment within families. Model 5 adjusted for model 4, plus physical activity, smoking total energy, alcohol, and sodium intake to adjust for behavioural risk factors. Model 6 included all factors included in model 5, plus psychosocial stress and depressive symptoms. Models 5 and 6 were not available for the Dutch population, as these data were not collected in this group. Secondly, the impact of BP-lowering medication on change in SBP was assessed, by fitting the linear regression models excluding participants using BP-lowering medication either at baseline or follow-up, and by running the analysis without adjustment for the use of BP-lowering medication in the levels of SBP but instead including use of BP-lowering medication as a covariate in the models. Lastly, we assessed the impact of missingness of covariates included in model 1–3 (missing for n = 174 participants) on the results of the regression analysis. Those with missing data on covariates differed significantly from those with complete data in terms of geographical location, follow-up time, level of education, and renal function. We therefore concluded missing values to be missing at random. We performed multiple imputation by chained equations using the ‘mice’ package for R (version 3.17.0), in which missing data is randomly imputed from a conditional distribution based on regression models including predictors of the missing variables.^14^ These predictors included the variables that were geographical location, age at baseline, follow-up time, baseline SBP and DBP, level of education, BMI, renal function, and diabetes mellitus, as well as hypertension at baseline, incident hypertension, delta SBP and DBP.^15^ Predictive mean matching method was applied for continuous variables, polytomous logistic regression for unordered categorical, proportional odds modelling for ordered categorical, and logistic regression for dichotomous variables, generating 20 imputed datasets. Subsequently, the linear and Poisson regression were run on the imputed datasets.

### Role of funding source

The funding source had no role in the design of the study, its execution, analyses, interpretation of the data, or decision to submit results.

## Results

### Population characteristics

A total of 4281 participants completed both baseline and follow-up data collection. After excluding 148 participants with missing data on BP, 4133 participants—1723 men and 2410 women—were included for analysis (Supplementary Figure S1), comprising 632 rural Ghanaian, 602 urban Ghanaian, 861 Amsterdam-Ghanaian, and 2038 Dutch participants.

58% of the study population was women, and the mean age was 46.5 years (standard deviation (SD) 12.2 years). Follow-up time was close to 7 years (mean 6.6 years, SD 1.01 years) for all groups and only slightly shorter in Dutch participants (Table 1).

**Table 1:** Baseline characteristics by geographical location and sex.

	Rural Ghana	Urban Ghana	Ghanaians in Amsterdam	Dutch	p-value	Missing (%)
**Men**						
n	223	178	342	980		
Follow-up time, years (mean (SD))	6·60 (0·46)	6·71 (0·38)	6·79 (0·86)	6·17 (1·20)	<0·001	0·2
Length of stay in Europe, years (median [IQR])	NA	NA	21·29 [14·69, 24·76]	NA	NA	82·3
Age, years (mean (SD))	48·22 (13·73)	46·77 (11·43)	48·47 (9·99)	46·77 (13·02)	0·097	0
Level of education (%)					<0·001	3·0
None/elementary	91 (43·5)	29 (17·2)	55 (17·1)	21 (2·2)		
Lower	82 (39·2)	82 (48·5)	140 (43·5)	89 (9·2)		
Intermediate	29 (13·9)	38 (22·5)	95 (29·5)	217 (22·3)		
Higher	7 (3·3)	20 (11·8)	32 (9·9)	644 (66·3)		
BMI, kg/m^2^ (mean (SD))	20·87 (2·77)	24·65 (4·21)	26·62 (3·46)	24·89 (3·38)	<0·001	0
Delta BMI, kg/m^2^ (mean (SD))	0·22 (1·86)	0·47 (2·36)	0·59 (1·70)	0·27 (1·60)	0·017	0·2
Diabetes mellitus (%)	8 (3·6)	13 (7·3)	38 (11·1)	32 (3·3)	<0·001	0·2
Waist-hip-ratio (mean (SD))	0·89 (0·06)	0·91 (0·07)	0·94 (0·06)	0·93 (0·07)	<0·001	0
eGFR, mL/min/1·73 m^2^ (mean (SD))	92·05 (17·07)	80·88 (14·46)	84·30 (16·02)	100·13 (14·17)	<0·001	1·1
Family history of hypertension	44 (19·8)	58 (33·0)	153 (48·7)	238 (35·7)	<0·001	20·0
Physical activity (%)					NA	70·6
High	143 (69·4)	111 (66·9)	85 (63·0)	NA		
Moderate	42 (20·4)	28 (16·9)	24 (17·8)	NA		
Low	21 (10·2)	27 (16·3)	26 (19·3)	NA		
Current smoking (%)	14 (6·7)	5 (3·0)	28 (8·9)	237 (24·3)	<0·001	3·1
Total energy intake, kcal/day (mean (SD))	2805 (976)	2388 (643)	2711 (941)	NA	<0·001	69·5
Sodium intake, mg/day (mean (SD))	2739 (1180)	3247 (1129)	2931 (898)	NA	<0·001	68·7
Alcohol intake, units/day (median [IQR])	0·03 [0·00, 0·20]	0·00 [0·00, 0·06]	0·00 [0·00, 0·37]	NA	<0·001	64·8
Psychosocial stress (%)	34 (16·5)	19 (11·2)	33 (10·5)	196 (20·1)	<0·001	3·4
Depressive symptoms (%)	9 (4·4)	1 (0·6)	22 (7·1)	46 (4·7)	0·016	3·6
Prescribed antihypertensive medication (%)	10 (4·5)	10 (5·6)	90 (26·3)	90 (9·2)	<0·001	0
**Women**						
n	409	424	519	1058		
Follow-up time, years (mean (SD))	6·62 (0·48)	6·72 (0·40)	6·79 (0·92)	6·24 (1·24)	<0·001	0·1
Length of stay in Europe, years (median [IQR])	NA	NA	19·75 [13·66, 24·00]	NA	NA	81·7
Age, years (mean (SD))	47·44 (12·82)	45·00 (10·55)	44·92 (9·15)	46·24 (13·45)	0·004	0
Level of education (%)					<0·001	3·0
None/elementary	270 (70·1)	193 (46·1)	192 (39·8)	23 (2·2)		
Lower	101 (26·2)	178 (42·5)	161 (33·4)	104 (9·9)		
Intermediate	8 (2·1)	38 (9·1)	105 (21·8)	206 (19·6)		
Higher	6 (1·6)	10 (2·4)	24 (5·0)	719 (68·3)		
BMI, kg/m^2^ (mean (SD))	23·70 (4.62)	28·68 (5·32)	29·43 (4·57)	23·87 (3·91)	<0·001	0·1
Delta BMI, kg/m^2^ (mean (SD))	0·84 (2·95)	1·07 (2·74)	1·37 (2·51)	0·42 (1·86)	<0·001	0·1
Diabetes mellitus (%)	23 (5·6)	32 (7·5)	48 (9·3)	20 (1·9)	<0·001	0·1
Waist-hip-ratio (mean (SD))	0·90 (0·07)	0·90 (0·06)	0·89 (0·07)	0·83 (0·07)	<0·001	0·1
eGFR, mL/min/1·73 m^2^ (mean (SD))	86·52 (18·13)	83·60 (16·37)	88·72 (17·54)	95·91 (14·26)	<0·001	1·1
Family history of hypertension	104 (25·6)	157 (37·5)	291 (60·5)	376 (48·8)	<0·001	13·9
Physical activity (%)					NA	58·3
High	211 (54·8)	193 (46·1)	127 (62·9)	NA		
Moderate	90 (23·4)	68 (16·2)	37 (18·3)	NA		
Low	84 (21·8)	158 (37·7)	38 (18·8)	NA		
Current smoking (%)	0 (0·0)	0 (0·0)	10 (2·1)	212 (20·1)	<0·001	3·3
Total energy intake, kcal/day (mean (SD))	2823 (1098)	2309 (649)	2457 (970)	NA	<0·001	57·8
Sodium intake, mg/day (mean (SD))	2752 (1198)	3135 (1098)	2897 (1007)	NA	<0·001	57
Alcohol intake, units/day (median [IQR])	0·00 [0·00, 0·03]	0·00 [0·00, 0·03]	0·00 [0·00, 0·07]	NA	0·016	52·8
Psychosocial stress (%)	85 (22·1)	59 (14·2)	86 (18·3)	283 (26·8)	<0·001	3·5
Depressive symptoms (%)	37 (9·6)	16 (3·9)	49 (10·5)	71 (6·7)	0·001	3·7
Prescribed antihypertensive medication (%)	38 (9·3)	56 (13·2)	132 (25·4)	83 (7·8)	<0·001	0

SD, standard deviation; IQR, interquartile range; BMI, body mass index; eGFR, estimated glomerular filtration rate; NA, not available.

Overall, men and women in rural Ghana had the most favourable cardiometabolic health profile and lifestyle with the lowest mean BMI, lowest diabetes mellitus prevalence, highest physical activity levels, lowest sodium intake, and they reported least often a family history of hypertension. In line with previously reported ethnic disparities in health, Ghanaians in Amsterdam were disadvantaged compared with the Dutch as they more often had elementary or lower levels of education, a higher mean BMI and BMI increase, higher diabetes mellitus prevalence, and were more likely to report a family history of hypertension. However, Dutch and participants in rural Ghana most often reported psychosocial stress, whereas depressive symptoms were most frequently present in Ghanaians in Amsterdam. In Ghanaians in Amsterdam, about one in four participants used BP-lowering medication, which was more frequent than in the other groups.

### Change in blood pressure

**Fig. 1:**
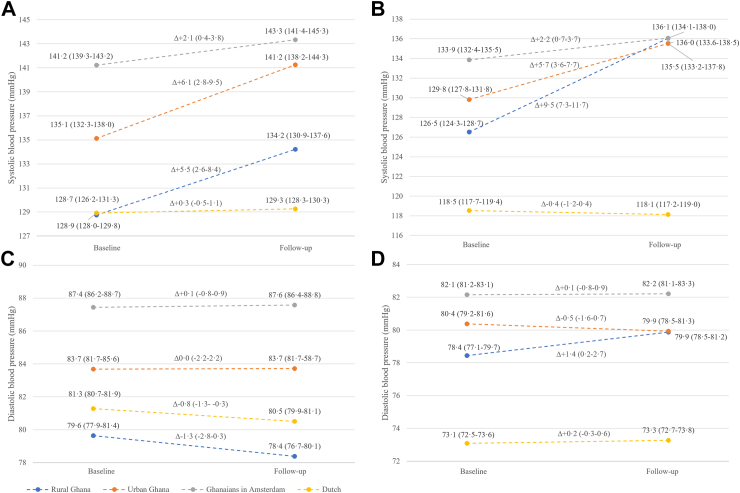
Mean age-standardised blood pressure at baseline and after 6·6 years of follow-up, per geographical location, in men (a systolic and c diastolic blood pressure) and women (b systolic and d diastolic blood pressure). Caption: Panel a and b display systolic blood pressure, c and d display diastolic blood pressure. Delta (Δ) indicates the crude change in blood pressure in mmHg between baseline and follow-up. For those on blood pressure lowering medication, systolic blood pressure was adjusted with +10 mmHg and diastolic blood pressure with +5 mmHg. Numbers between brackets indicate are 95% confidence intervals.

Mean age-standardised SBP and DBP were highest in Amsterdam-Ghanaian, in both sexes and at both time points (Fig. 1). In men, increase in SBP was largest in urban Ghana (+6·1 mmHg, 95% confidence interval (95% CI) 2·8–9·5 mmHg), followed by rural Ghana (+5·5 mmHg, 95% CI 2·6–8·4 mmHg), whereas the increase in SBP was less pronounced in Ghanaians in Amsterdam (+2·1 mmHg, 95% CI 0·4–3·8 mmHg). In women, the largest age-standardised increase in SBP occurred in rural Ghana (+9·5 mmHg, 95% CI 7·3–11·7 mmHg), bringing the mean SBP at follow-up at par with the SBP of Ghanaian women in Amsterdam: 136·0 mmHg (95% CI 133·6–138·5 mmHg) in rural Ghana compared to 136·1 mmHg (95% CI 134·1–138·0 mmHg) in Amsterdam. SBP remained stable over time in Dutch, in both sexes. DBP remained stable over time in most groups but showed a 1·3 mmHg (95% CI −2·8 to 0·3 mmHg) decrease in men in rural Ghana, a 0·8 mmHg (95% CI −1·3 to 0·3 mmHg) decrease in Dutch men, and a 1·4 mmHg (95% CI 0·2–2·7 mmHg) increase in women in rural Ghana.

**Fig. 2:**
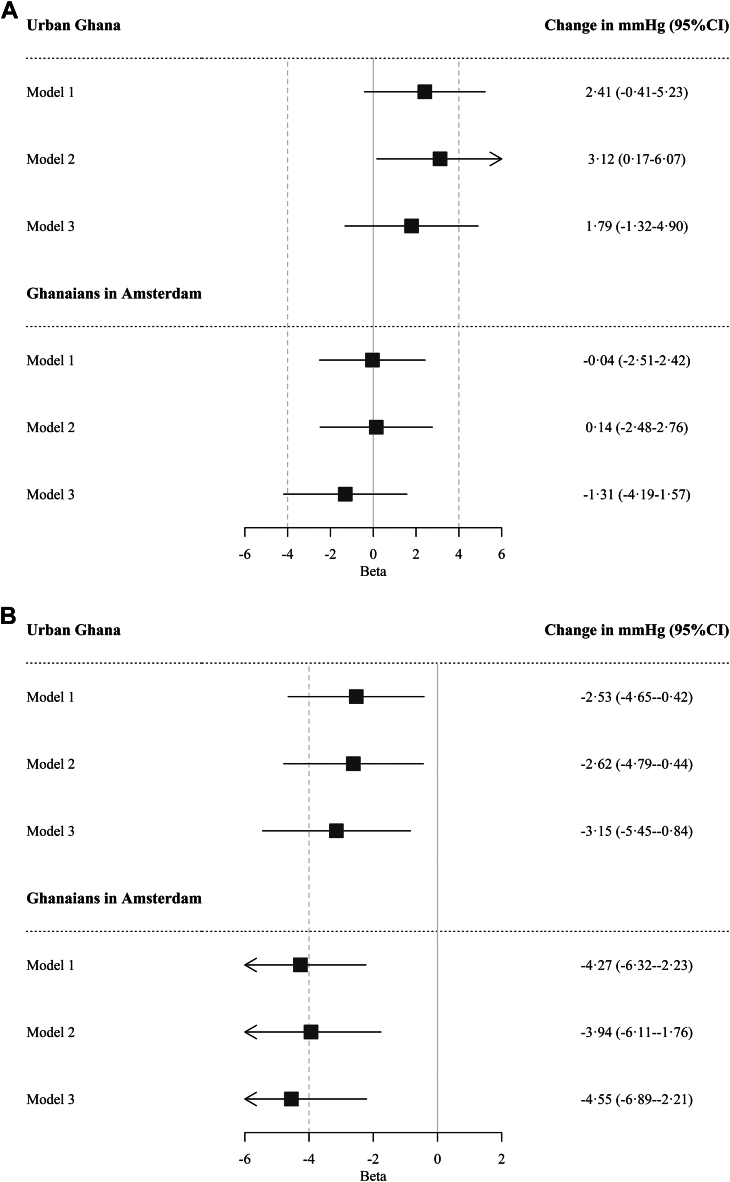
Change in 6·6 years in systolic blood pressure (mmHg) in Ghanaians in urban Ghana and Amsterdam relative to rural Ghana, in men (A) and women (B). Caption: Model 1 is adjusted for age at baseline, follow-up time, and baseline systolic blood pressure; model 2 is adjusted for model 1 plus education; model 3 is adjusted for model 2 plus body mass index, estimated glomerular filtration rate, and diabetes mellitus. CI, confidence interval.

Compared to men in rural Ghana (Fig. 2), change in SBP was larger in men in urban Ghana (3·12 mmHg change, 95% CI 0·17–6·07 mmHg, model 2), but these differences disappeared after adjustment for BMI, eGFR and diabetes status (1·79 mmHg chance, 95% CI −1·32 to 4·90 mmHg, model 3). There was no difference in change in SBP between Ghanaians in rural Ghana and Amsterdam (−1·31 mmHg chance, 95% CI −4·19 to 1·57 mmHg). For Ghanaian women, compared to rural Ghana, delta SBP was smaller in urban Ghana (−3·15 mmHg change, 95% CI −5·45 to 0·84 mmHg, model 3) and Amsterdam (−4·55 mmHg change, 95% CI −6·89 to 2·21 mmHg, model 3), and these differences were independent of adjustment for covariates.

**Fig. 3:**
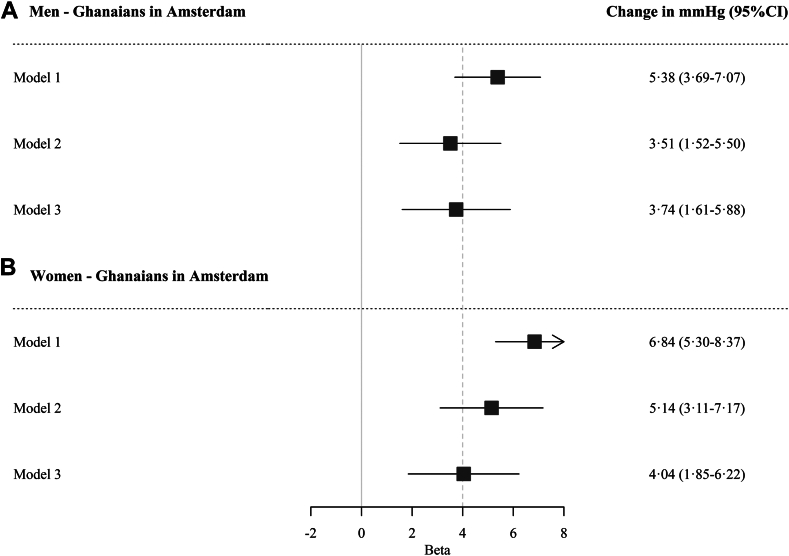
Change in systolic blood pressure (mmHg) during 6·6 years in Ghanaians in Amsterdam relative to Dutch, in men (a) and women (b). Caption: Model 1 is adjusted for age at baseline, follow-up time, and baseline systolic blood pressure; model 2 is adjusted for model 1 plus education; model 3 is adjusted for model 2 plus body mass index, estimated glomerular filtration rate, and diabetes mellitus. CI, confidence interval.

Compared to the Dutch (Fig. 3), both Ghanaian men (3·74 mmHg change, 95% CI 1·61–5·88 mmHg) and women (4·04 mmHg change, 95% CI 1·85–6·22 mmHg) in Amsterdam had a significantly larger change in SBP, and this difference persisted after adjustment for conventional CVD risk factors.

Sensitivity analysis including additional covariates to the analysis (model 2b–6) did not change the findings in men (Supplementary Table S1). After adjustment for behavioural factors (model 5), change in SBP no longer differed between women in rural Ghana, urban Ghana, and Amsterdam. In both men and women, compared to the Dutch, change in SBP was persistently larger in Ghanaians in Amsterdam, even after adjustment for additional covariates (Supplementary Table S2).

Excluding those on BP-lowering medication or removing the adjustment for the use of BP-lowering medication did not affect the results (Supplementary Tables S3 and S4). Running model 1–3 of the regression analysis on the imputed datasets did not significantly affect the results (Supplementary Table S7).

### Prevalence and incidence of hypertension

**Fig. 4:**
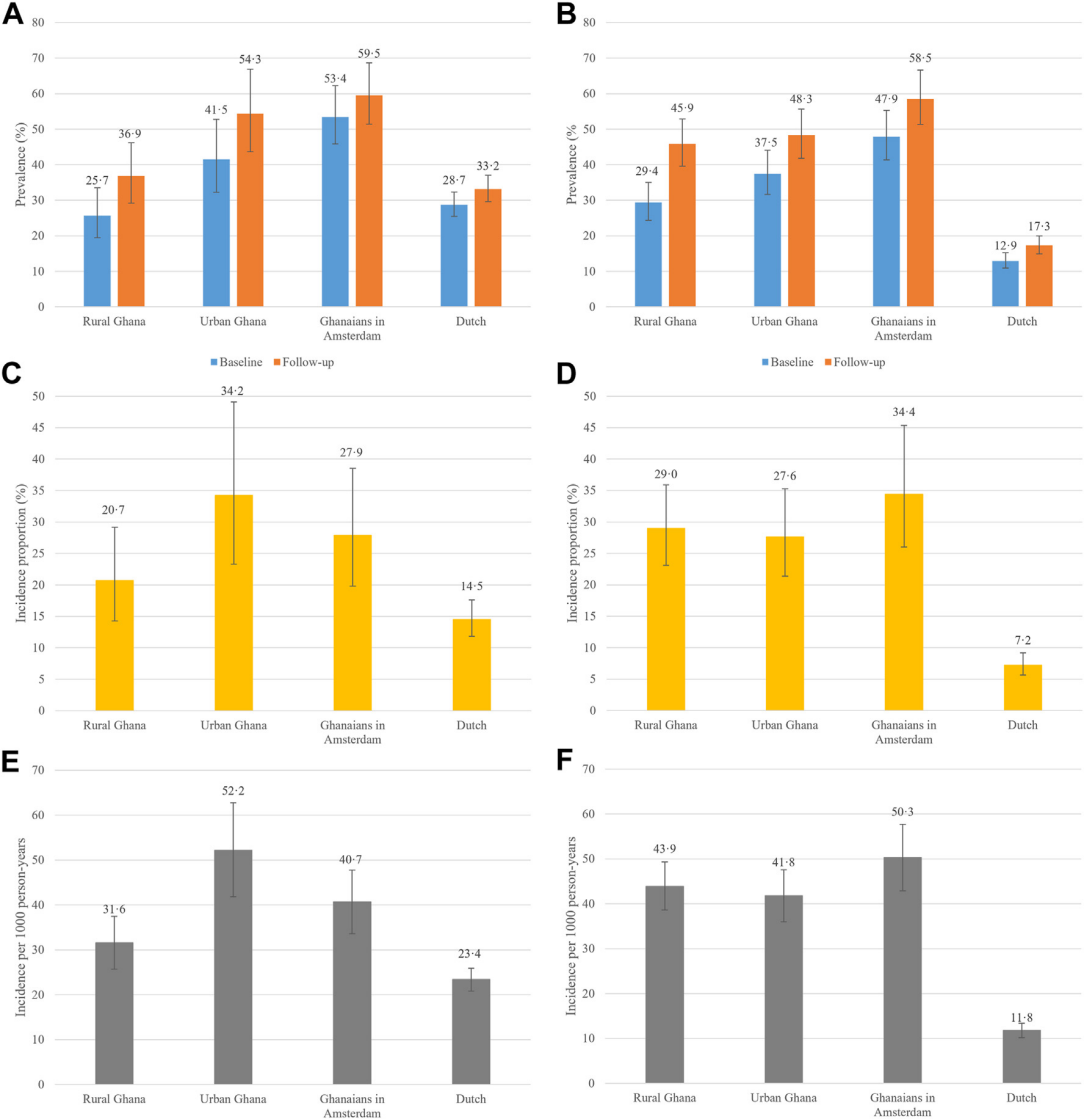
Age-standardised prevalence of hypertension at baseline and follow-up, by geographical location in men (a) and women (b); age-standardised 6-years incidence proportion of hypertension, by geographical location for men (c) and women (d); and hypertension incidence per 1000 person-years in men (e) and women (f). Caption: Error bars are 95% confidence intervals.

Hypertension prevalence and incidence across populations and by sex are shown in Fig. 4. Hypertension prevalence increased over time, in both men and women and in all geographical locations. In men, at follow-up, age-standardised hypertension prevalence remained highest in Ghanaians in Amsterdam (59·5%, 95% CI 51·5–68·7%), closely followed by Ghanaians in urban Ghana (54·4%, 95% CI 43·7–66·9%) and was lowest in Dutch (33·2%, 95% CI 29·6–37·0%). At follow-up, the prevalence of hypertension among women in rural Ghana was approaching the prevalence among women in urban Ghana, namely 45·9% (95% CI 39·6–52·9%) vs. 48·3% (95% CI 41·8–55·7%), respectively. Prevalence of hypertension was highest in Ghanaian women in Amsterdam (58·5%, 95% CI 51·3–66·6%) and lowest in Dutch women (17·3%, 95% CI 14·9–20·0%).

Age-standardised hypertension incidence proportions were highest in men in urban Ghana (34·2%, 95% CI 23·3–49·1%) and Ghanaian women in Amsterdam (34·4%, 95% CI 26·0–45·4%). In women, the incidence proportion was slightly higher in rural Ghana (29·0%, 95% CI 23·1–35·9%) than in urban Ghana (27·6%, 95% CI 21·4–35·3%). Incidence proportions were lowest in Dutch men (14·5%, 95% CI 11·8–17·6%) and women (7·2%, 95% CI 5·6–9·2%), which was reflected in the incidence per 1000 person-years.

Compared to rural Ghana, the hypertension incidence rate ratios (Supplementary Figure S2) did not differ in urban Ghana (IRR 1·09, 95% CI 0·86–1·39 in men, IRR 0·98, 95% CI 0·83–1·15 in women) or Ghanaians in Amsterdam (IRR 1·01, 95% CI 0·80–1·28 in men, IRR 0·98, 95% CI 0·82–1·16 in women), and adjustment for conventional risk factors did not alter the differences in both men and women. Compared to the Dutch (Supplementary Figure S3), the hypertension incidence rate ratio was similar in Ghanaian men in Amsterdam (IRR 1·03, 95% CI 0·82–1·28). Ghanaian women in Amsterdam had a 1·14 times higher incidence rate (95% CI 1·00–1·30) compared to Dutch women, but this difference disappeared after adjustment for the level of education (IRR 1·12, 95% CI 0·94–1·34).

The sensitivity analyses with additional variables did not impact the results (Supplementary Tables S5 and S6). Imputation of missing covariates included in model 1–3 of the regression analyses did not significantly affect the results of the Poisson regression (Supplementary Table S7).

## Discussion

Between baseline and follow-up, SBP increased in the Ghanaian populations residing in different geographical locations, especially in rural and urban Ghana. Compared to rural Ghana, men in urban Ghana experienced the largest rise in SBP, influenced by BMI, eGFR, and diabetes, while Ghanaian women in urban Ghana and Amsterdam showed smaller increases compared to their rural counterparts. The SBP change in Ghanaians in Amsterdam was significantly larger than in Dutch for both men and women, regardless of adjustments. Hypertension incidence was highest in men in urban Ghana and Ghanaian women in Amsterdam, while the lowest rates were observed in Dutch women. There was no significant difference in hypertension incidence between Ghanaians in rural Ghana and other geographical locations, and between Ghanaians in Amsterdam and Dutch participants.

This study findings show that mean SBP and hypertension prevalence rates among people in rural Ghana have converged towards those observed in urban and Ghanaians based in Amsterdam. This follows trends in SSA, showing that BP and hypertension prevalence have increased in the past decades.^2^ It is unclear, however, to what extent this increase affects both urban and rural areas in SSA. Our results show that the urban lifestyle and associated cardiovascular risk profile has reached rural areas in SSA. In rural and urban Ghana, hypertension prevalence increased by more than 10% over a period of 6·6 years, to 43% in rural Ghana and 51% in urban Ghana at follow-up. This high prevalence of hypertension is in line with findings in other SSA middle-income countries like Nigeria^16^^,^^17^ and South Africa.^18^ Additionally, findings from a previous systematic review meta-analysing the hypertension prevalence among people aged >50 years in SSA, reported a hypertension prevalence of 53%.^19^ Also, they reported an increase of hypertension prevalence over time, from 51% in the period 2005–2009 to 62% in the period 2015–2019,^19^ which corroborates our findings. Moreover, we found a 6-years hypertension incidence of 29% (1-years incidence 4·4%) in the Ghanaian populations in different locations, with highest incidence in men in urban Ghana and Ghanaian women in Amsterdam. Population-based longitudinal studies describing hypertension incidence in SSA population are scarce, but a 5-years prospective study among Black South Africans aged >30 years found a comparable hypertension incidence of 24% (1-year incidence 4·8%) amongst those with a BP of ≤120/80 mmHg at baseline,^20^ and progression was mainly attributable to alcohol intake and waist circumference. Another population-based study from rural South Africa, showed hypertension incidence rates among those aged >40 years to be 8·37 per 100 person-years, which is higher compared to the incidence in rural Ghana reported in our study.^21^ Older age, being employed, and having a high waist circumference were associated with higher risk of incident hypertension.^21^ In our study, compared to participant with incident hypertension, participants who remained normotensive (BP < 140/90 mmHg, no medication) were generally younger, had a higher level of education, were more frequently smokers, had a higher total energy intake, but had a lower BMI and waist circumference, less diabetes mellitus, higher eGFR, and SBP and DBP remained stable over the years, with similar patterns across the geographical locations. This suggests that preventative efforts should focus on younger populations to prevent them from developing hypertension, although further research into the determinants of incident hypertension in this cohort is needed.

Women in rural Ghana, especially, had a significantly larger increase in SBP than women in the other geographical locations. These differences persisted after adjustment for hypertension risk factors associated with urbanisation,^8^ which was in contrast to our hypothesis. In particular, change in BMI—being a strong determinant of BP^13^—did not explain the difference between geographical locations. On one hand, this suggests that women in rural settings have similar hypertension risk factor profile to women in urban Ghana and Amsterdam, based on the factors included in our models, and therefore, do not explain the geographical differences. On the other hand, it implies that unobserved or unmeasured factors may be influencing SBP differently in women in rural Ghana, compared to the other groups, accounting for the geographical differences. For instance, menopause is known to impact SBP, and studies have shown that age of menopause can differ between geographical locations.^22^ It is unclear, however, whether there are differences in age of menopause between rural and urban Ghana, and whether this would impact SBP differently between locations. Salt sensitivity increases after menopause.^23^ Rural-urban differences in salt intake interacting with menopausal status and BMI could contribute to the observed differences. Additionally, early life factors such as maternal malnutrition during pregnancy, or severe acute malnutrition in childhood have shown to be associated with BP increase and hypertension during adulthood,^24^ which might be a contributing factor to the different BP trajectories between rural and urban settings. Thus, further research into pathophysiological mechanisms driving the large change in BP in rural and urban Ghana is needed.

In Amsterdam, The Netherlands, age-standardised hypertension incidence proportion was about twice as high in Ghanaian men and four times higher in Ghanaian women compared to Dutch men and women. This ethnic difference in hypertension incidence is in line with previous population-based longitudinal studies from the USA, showing an incidence rate that was twice as high in African Americans as in white Americans.^25^ Also in a South African cohort study, hypertension prevalence increased over time in Black South Africans but not in white South Africans.^26^ The differences in BP and incident hypertension between Ghanaians in Amsterdam and Dutch were not explained by conventional risk factors, suggesting the need for more research into other potential factors, including system level factors, that may explain the observed differences. For instance, even though substantial efforts have been made to increase the awareness of risk factor for hypertension among the Ghanaian communities in Amsterdam,^27^ this has not translated in diminishing the “SBP gap” between the Dutch and Ghanaian population. The high burden of hypertension in Ghanaians asks for urgent interventions that go beyond hypertension awareness campaigns.

In 2013, the WHO set the goal to reduce the prevalence of hypertension by 25% in 2025.^28^ In this light, the Ghanaian government has developed several health policies working towards achieving this goal, such as the NCD policy^29^ and the Universal Health Coverage roadmap.^30^ A recent review of these policies showed that these plans have low levels of integration of hypertension prevention and control across most health policies.^31^ Specifically, poor financial allocation, low task-sharing initiatives, and lack of involvement of public and patients in policymaking, will hamper the efforts of reaching the WHO 2025 goals.^31^ This, in combination with the observed high and increasing prevalence of hypertension in both rural and urban settings in our study, requests for critical reflection on the organisation of hypertension preventative efforts.^32^

In The Netherlands, “health in all policies” approach is aiming to reduce inequalities in health.^33^ In this setting, there is growing attention for ethnicity as a driver of health inequalities, and increasing voices to take ethnicity into account in health policies and CVD screening.^34^ Given the large and increasing disparity in the burden of hypertension between the Dutch and Ghanaian population in our study, more action needs to be taken to ensure that hypertension preventative measures are reaching all inhabitants of The Netherlands. This approach should go beyond the individual level lifestyle advice, but should assess system level factors, such as neighbourhood urban design and food environment, contributing to the disparities in hypertension burden.

For future research, studies into the effects of this high burden of hypertension in different geographical settings should be assessed. Additionally, effective population-specific interventions should be researched and developed, such as intervening in socio-cultural environment and community approaches, to curb the rising trend in hypertension. Moreover, longitudinal population-based cohort studies remain scarce in the SSA context, as do longitudinal multi-ethnic studies in Europe. Investments should be made to develop these cohorts,^35^ as these cohorts are urgently needed to evaluate time trends and drivers of disease.

A major strength of this study is the use of longitudinal data from a multi-centre cohort study including participants residing in rural and urban Ghana, Ghanaians in The Netherlands, and a Dutch population. Data collection was highly standardised, ensuring comparability over time and between the different populations.

Our study has several limitations. The response and participation rates differed substantially between the populations and were low for follow-up in urban Ghana and Ghanaians in Amsterdam, due to the COVID-19 pandemic, which started in the middle of data collection. Non-response analysis showed that those lost to follow-up did not differ in sex, level of education or BMI, but were more often hypertensive or had diabetes at baseline. Thus, our data might underestimate the prevalence of hypertension. The method of contacting eligible participants differed between the geographical locations, and was chosen based on what was deemed most appropriate for each specific geographical location. However, this approach may have influenced the response rate. For example, in urban Ghana, changes in telephone numbers over time occasionally hindered the research team's ability to reach participants, which is reflected in the lower response rate in this setting. The data collection itself, however, was conducted in person, so the mode of contact did not impact the accuracy of the measurements between the geographical locations. Additionally, we excluded 148 participants from our analysis because of missing BP data. These excluded participants were more often Ghanaians in Amsterdam, of younger age but with higher BMI at baseline, potentially affecting our observations. Imputation of missing covariates included in model 1–3 of the regression analyses did not significantly affect the results. Moreover, comparing participants with data collected before and after the first reported case of COVID-19 revealed the following: among Ghanaians in Amsterdam, those examined after the onset of the pandemic were less often men, slightly younger (46·9 years before vs. 45·6 years after start of the pandemic), had a longer follow-up period (6·6 years vs. 7·0 years), and showed a somewhat larger change in both systolic (+0·5 mmHg vs. +4·1 mmHg) and diastolic blood pressure (−1·3 vs. +1·0 mmHg). However, there was no difference in the incidence of hypertension. This suggests that the COVID-19 pandemic might have impacted our findings. Lastly, BP was measured at least twice at a single occasion, which could have falsely increased the BP readings, for instance due to the white coat effect. However, as the prevalence of white coat hypertension is expected to be equal between the various geographical locations and ethnic populations, comparisons between the groups remain valid.

In this longitudinal study across different geographical locations, we show that increases in SBP and hypertension prevalence are more pronounced in urban and rural Ghana compared to Ghanaians living in The Netherlands. Although the absolute hypertension prevalence rates were higher in urban Ghana, our data suggest that the SBP increase and hypertension prevalence in rural Ghana is catching up - especially in women - suggesting that urban lifestyle and associated cardiovascular risk factors are also reaching rural communities. This large change in SBP and high burden of hypertension in migrants and non-migrants from Ghana calls for preventative measures to curb the rise in hypertension incidence, and its related CVD complications.

## Contributors

CA, EOD, BJB, and ELL were responsible for the conceptualisation of this study. EB and SND were responsible for project administration, recruitment and data collection, alongside ELL, KACM, CA, and EOD. All authors had access to the data. STA, ELL, and KACM were involved in data curation and verified the underlying data reported in the manuscript. Formal analysis were performed by ELL, supervised by MHB, BJB, and CA. ELL wrote the original draft, and all authors were involved in reviewing and editing of the manuscript. All authors read and approved the final version of the article.

## Data sharing statement

Data will be available upon reasonable request with the RODAM-Pros study coordinator. They can be reached at rodamcoordinator@amsterdamumc.nl.

## Declaration of interests

None declared.
