# Supplementary material for: Blood pressure change and hypertension incidence among Ghanaians living in rural Ghana, urban Ghana and The Netherlands: a prospective cohort study
Source: eClinicalMedicine. 2025 Mar 5;81:103141. doi: 10.1016/j.eclinm.2025.103141 (PMC11925589; doi:10.1016/j.eclinm.2025.103141)
Supplement: Supplementary Materials [file mmc1.docx]

**Supplementary files**

**Table of contents**

| Section | Page |
| --- | --- |
| Supplementary File 1 – Measurements and definitions | 2 |
| Supplementary Table 1 – Change in systolic blood pressure in Ghanaians in urban Ghana and Amsterdam relative to rural Ghana, in men and women, with adjustment for additional covariates | 4 |
| Supplementary Table 2 – Change in systolic blood pressure in Ghanaians in Amsterdam relative to Dutch, in men and women, with adjustment for additional covariates | 5 |
| Supplementary Table 3 – Change in systolic blood pressure in Ghanaians in urban Ghana and Amsterdam relative to rural Ghana, in men and women, excluding those on blood pressure lowering medication | 6 |
| Supplementary Table 4 – Change in systolic blood pressure in Ghanaians in urban Ghana and Amsterdam relative to rural Ghana, in men and women, excluding those on blood pressure lowering medication | 6 |
| Supplementary Table 5 – Incident rate ratio for hypertension in Ghanaians in urban Ghana and Amsterdam relative to rural Ghana, in men and women, with adjustment for additional covariates | 7 |
| Supplementary Table 6 – Incident rate ratio for hypertension in Ghanaians in Amsterdam relative to Dutch, in men and women, with adjustment for additional covariates | 8 |
| Supplementary Table 7 – Incident rate ratio for hypertension and change in systolic blood pressure after multiple imputation of missing covariates, in Ghanaians in urban Ghana and Amsterdam relative to rural Ghana, and in Ghanaians in Amsterdam relative to Dutch | 9 |
| Supplementary Figure 1 – Flowchart of participants included in the analyses | 10 |
| Supplementary Figure 2 – Incident rate ratio for in Ghanaians in urban Ghana and Amsterdam relative to rural Ghana, in men (A) and women (B) amongst those without hypertension at baseline | 11 |
| Supplementary Figure 3 – Incident rate ratio for hypertension in Ghanaians in Amsterdam relative to Dutch, in men (A) and women (B) amongst those without hypertension at baseline | 12 |

**Supplementary File 1 – Measurements and definitions**

Standardised protocols were used for data collection, to ensure comparability of the results between the different study populations and over time. Data were collected by trained research assistants.

During a structured, in-person interview performed by trained ethnically matched research personnel, data on demographics, socioeconomic status (SES), medical history and behavioural factors were collected using health questionnaires. Dutch participants completed an online digital Dutch version of the structured health questionnaire.

Level of education was used as a proxy for SES, based on the highest education attainment obtained in Ghana or the Netherlands. Education was classified into no or elementary, lower, intermediate and higher education. Family history of hypertension was assessed by asking whether any first degree relative had been diagnosed with high BP. The WHO (World Health Organization) Global Physical Activity Questionnaire (GPAQ) V2.0 was used to ask participants about the amount of physical activity at work, at home and while commuting.^1^ This was subsequently quantified in metabolic equivalents (METs), and classified into lower, intermediate and higher level of physical activity. Smoking was grouped into those who were current smokers and former/never smokers. Total daily energy intake (kcal/day) was calculated based on the Ghana-specific Food Propensity Questionnaire (FPQ),^2^ adapted from the European FPQ. Alcohol and sodium intake were calculated from the FPQ and quantified in units per day and mg per day, respectively. The stress scale used in the INTERHEART study were used to assess stress at work and at home, and was classified into no (never experienced stress at home and/or work) or yes (experienced periods of stress at home and/or work).^3^ Depressed mood was defined as having a score of at least 10 on the Patient Health Questionnaire 9 (PHQ-9), a questionnaire assessing the prevalence of depressive symptoms over the preceding 2 weeks.^4^

During physical examination, anthropometric measures and blood pressure measurements were taken at least twice, and the mean of the first two readings were used for analyses. Weight was measured in light clothing, without shoes on a SECA 877 weighing scale. Height was recorded using a portable SECA 217 stadiometer. Body mass index (BMI) was calculated by dividing height in meters squared by weight in kilograms (kg/m^2^). Waist circumference was measured in centimetres at the midpoint between the lower rib and the iliac crest. Hip circumference was measured over the trochanter major. Waist-to-hip-ratio (WHR) was calculated by dividing the waist by the hip circumference. Using a validated semiautomated device (WatchBP Home, Microlife AG Swiss Corporation, Widnau, Switzerland), BP was measured in a sitting position, after at least five minutes rest, using an appropriate cuff size on the left upper arm. Participants were instructed to bring their prescribed medication to the research location, and medication names, dosages and frequency of use were recorded. Medication was subsequently grouped based on the Anatomical Therapeutic Chemical (ATC) classification.^5^

Hypertension was defined as having a systolic BP (SBP) of ≥140 mmHg or a diastolic BP (DBP) of ≥90 mmHg or the use of antihypertensive medication as classified by ATC code. Incident hypertension was defined as the presence of hypertension at follow-up in the absence of hypertension at baseline.

Venous blood samples were collected after an overnight fast of at least ten hours. Fasting plasma glucose was measured using a hexokinase spectrophotometric measurement. Diabetes mellitus was defined based on self-reported diagnosis of diabetes, on fasting plasma glucose at least 7 mmol/L and/or use of glucose-lowering medication (based on ATC coding).^6^ Plasma creatinine was determined with an enzymatic colorimetric rest. Kidney function was estimated using the estimated glomerular filtration rate (eGFR), without adjustment for ethnic origin (CKD-EPI 2021).^7,8^

**References**

1. Bull FC, Maslin TS, Armstrong T. Global physical activity questionnaire (GPAQ): nine country reliability and validity study. J Phys Act Health. 2009;6(6):790-804.

2. Galbete C, Nicolaou M, Meeks KA, de-Graft Aikins A, Addo J, Amoah SK, et al. Food consumption, nutrient intake, and dietary patterns in Ghanaian migrants in Europe and their compatriots in Ghana. Food Nutr Res. 2017;61(1):1341809.

3. Yusuf S, Hawken S, Ounpuu S, Dans T, Avezum A, Lanas F, et al. Effect of potentially modifiable risk factors associated with myocardial infarction in 52 countries (the INTERHEART study): case-control study. Lancet. 2004;364(9438):937-52.

4. Kroenke K, Spitzer RL, Williams JB, Lowe B. The Patient Health Questionnaire Somatic, Anxiety, and Depressive Symptom Scales: a systematic review. Gen Hosp Psychiatry. 2010;32(4):345-59.

5. WHO Collaborating Centre for Drug Statistics Methodology. Guidelines for ATC classiciation and DDD assignement, 2021. 2022.

6. World Health Organization. Definition and diagnosis of diabetes mellitus and intermediate hyperglycemia: report of a WHO/IDF consultation. Geneva: WHO; 2006.

7. Inker LA, Eneanya ND, Coresh J, Tighiouart H, Wang D, Sang Y, et al. New Creatinine- and Cystatin C-Based Equations to Estimate GFR without Race. N Engl J Med. 2021;385(19):1737-49.

8. Miller WG, Kaufman HW, Levey AS, Straseski JA, Wilhelms KW, Yu HE, et al. National Kidney Foundation Laboratory Engagement Working Group Recommendations for Implementing the CKD-EPI 2021 Race-Free Equations for Estimated Glomerular Filtration Rate: Practical Guidance for Clinical Laboratories. Clin Chem. 2022;68(4):511-20.

**Supplementary Table 1** – **Change in systolic blood pressure in Ghanaians in urban Ghana and Amsterdam relative to rural Ghana, in men and women, with adjustment for additional covariates**

| Men |  |  | Women |  |  |
| --- | --- | --- | --- | --- | --- |
| *Model 2* | *Change (mmHg) (95%CI)* | *p-value* | ***Model 2*** | *Change (mmHg) (95%CI)* | *p-value* |
| Rural Ghana | Ref. |  | Rural Ghana | Ref. |  |
| Urban Ghana | 3·12 (0·17-6·07) | 0·04 | Urban Ghana | -2·62 (-4·79--0·44) | 0·02 |
| Ghanaians in Amsterdam | 0·14 (-2·48-2·76) | 0·92 | Ghanaians in Amsterdam | -3·94 (-6·11- -1·76) | <0·001 |
| *Model 2b* | *Change (mmHg) (95%CI)* | *p-value* | ***Model 2b*** | *Change (mmHg) (95%CI)* | *p-value* |
| Rural Ghana | Ref. |  | Rural Ghana | Ref. |  |
| Urban Ghana | 2·85 (-0·09- 5·77) | 0·06 | Urban Ghana | -2·85 (-5·01- -0·69) | 0·001 |
| Ghanaians in Amsterdam | -0·36 (-2·97- 2·24) | 0·78 | Ghanaians in Amsterdam | -4·46 (-6·63- -2·3) | <0·001 |
| *Model 3* | *Change (mmHg) (95%CI)* | *p-value* | ***Model 3*** | *Change (mmHg) (95%CI)* | *p-value* |
| Rural Ghana | Ref. |  | Rural Ghana | Ref. | 0·01 |
| Urban Ghana | 1·79 (-1·51-5·08) | 0·29 | Urban Ghana | -3·15 (-5·45- -0·84) | <0·001 |
| Ghanaians in Amsterdam | -1·31 (-4·19-1·57) | 0·37 | Ghanaians in Amsterdam | -4·55 (-6·89- -2·21) | <0·001 |
| *Model 3b* | *Change (mmHg) (95%CI)* | *p-value* | ***Model 3b*** | *Change (mmHg) (95%CI)* | *p-value* |
| Rural Ghana | Ref. |  | Rural Ghana | Ref. |  |
| Urban Ghana | 1·86 (-1·25- 4·97) | 0·24 | Urban Ghana | -2·82 (-5·13- -0·51) | 0·02 |
| Ghanaians in Amsterdam | -1·26 (-4·14- 1·61) | 0·39 | Ghanaians in Amsterdam | -3·85 (-6·22- -1·48) | 0·001 |
| *Model 4* | *Change (mmHg) (95%CI)* | *p-value* | ***Model 4*** | *Change (mmHg) (95%CI)* | *p-value* |
| Rural Ghana | Ref. |  | Rural Ghana | Ref. |  |
| Urban Ghana | 1·78 (-1·51- 5·08) | 0·29 | Urban Ghana | -3·06 (-5·46- -0·66) | 0·01 |
| Ghanaians in Amsterdam | -1·33 (-4·45- 1·78) | 0·40 | Ghanaians in Amsterdam | -4·38 (-6·88- -1·87) | <0·001 |
| *Model 5* | *Change (mmHg) (95%CI)* | *p-value* | ***Model 5*** | *Change (mmHg) (95%CI)* | *p-value* |
| Rural Ghana | Ref. |  | Rural Ghana | Ref. |  |
| Urban Ghana | 3·61 (-1·64- 8·85) | 0·18 | Urban Ghana | -2·32 (-5·82- 1·17) | 0·19 |
| Ghanaians in Amsterdam | 0·43 (-5·39- 6·26) | 0·88 | Ghanaians in Amsterdam | -2·42 (-6·62- 1·78) | 0·26 |
| *Model 6* | *Change (mmHg) (95%CI)* | *p-value* | ***Model 6*** | *Change (mmHg) (95%CI)* | *p-value* |
| Rural Ghana | Ref. |  | Rural Ghana | Ref. |  |
| Urban Ghana | 3·61 (-1·68- 8·90) | 0·18 | Urban Ghana | -2·32 (-5·84- 1·20) | 0·20 |
| Ghanaians in Amsterdam | -0·25 (-6·20- 5·71) | 0·94 | Ghanaians in Amsterdam | -2·92 (-7·18- 1·35) | 0·18 |

*CI, confidence interval.*

*Model 2 adjusted for is adjusted for age, follow-up time, baseline SBP (plus 10 mmHg for those on blood pressure lowering medication), level of education;*

*Model 2b adjusted for model 2 plus delta BMI;*

*Model 3 adjusted for model 2 plus BMI, eGFR, diabetes mellitus at baseline;*

*Model 3b adjusted for model 3 plus and waist-to-hip ratio;*

*Model 4 adjusted for model 3 plus family history of hypertension;*

*Model 5 adjusted for model 4 plus physical activity, total energy intake, smoking, alcohol intake and sodium intake;*

*Model 6 adjusted for model 5 plus psychosocial stress and depression.*

**Supplementary Table 2** – **Change in systolic blood pressure in Ghanaians in Amsterdam relative to Dutch, in men and women, with adjustment for additional covariates**

| Men |  |  | Women |  |  |
| --- | --- | --- | --- | --- | --- |
| *Model 2* | Change (mmHg) (95%CI) | p-value | ***Model 2*** | Change (mmHg) (95%CI) | p-value |
| Dutch | Ref. |  | Dutch | Ref. |  |
| Ghanaians in Amsterdam | 3·51 (1·52- 5·50) | <0·001 | Ghanaians in Amsterdam | 5·14 (3·11- 7·17) | <0·001 |
| *Model 2b* | Change (mmHg) (95%CI) | p-value | ***Model 2b*** | Change (mmHg) (95%CI) | p-value |
| Dutch | Ref. |  | Dutch | Ref. |  |
| Ghanaians in Amsterdam | 3·19 (1·23-5·14) | 0·001 | Ghanaians in Amsterdam | 4·42 (2·40-6·44) | <0·001 |
| *Model 3* | Change (mmHg) (95%CI) | p-value | ***Model 3*** | Change (mmHg) (95%CI) | p-value |
| Dutch | Ref. |  | Dutch | Ref. |  |
| Ghanaians in Amsterdam | 3·74 (1·61-5·88) | 0·001 | Ghanaians in Amsterdam | 4·04 (1·85-6·22) | <0·001 |
| *Model 3b* | Change (mmHg) (95%CI) | p-value | ***Model 3b*** | Change (mmHg) (95%CI) | p-value |
| Dutch | Ref. |  | Dutch | Ref. |  |
| Ghanaians in Amsterdam | 4·16 (2·00-6·33) | <0·001 | Ghanaians in Amsterdam | 3·91 (1·73-6·09) | <0·001 |
| *Model 4* | Change (mmHg) (95%CI) | p-value | ***Model 4*** | Change (mmHg) (95%CI) | p-value |
| Dutch | Ref. |  | Dutch | Ref. |  |
| Ghanaians in Amsterdam | 3·78 (1·22-6·35) | 0·003 | Ghanaians in Amsterdam | 4·59 (2·01;7·17) | <0·001 |

*CI, confidence interval.*

*Model 2 adjusted for is adjusted for age, follow-up time, baseline SBP (plus 10 mmHg for those on blood pressure lowering medication), level of education;*

*Model 2b adjusted for model 2 plus delta BMI;*

*Model 3 adjusted for model 2 plus BMI, eGFR, diabetes mellitus at baseline;*

*Model 3b adjusted for model 3 plus and waist-to-hip ratio;*

*Model 4 adjusted for model 3 plus family history of hypertension.*

**Supplementary Table 3 – Change in systolic blood pressure in Ghanaians in urban Ghana and Amsterdam relative to rural Ghana, in men and women, excluding those on blood pressure lowering medication**

| Men |  |  | Women |  |  |
| --- | --- | --- | --- | --- | --- |
| *Model 1* | *Change (mmHg) (95%CI)* | *p-value* | ***Model 1*** | *Change (mmHg) (95%CI)* | *p-value* |
| Rural Ghana | Ref. |  | Rural Ghana | Ref. |  |
| Urban Ghana | 1·02 (-1·87- 3·91) | 0·49 | Urban Ghana | -3·30 (-5·33- -1·16) | 0·002 |
| Ghanaians in Amsterdam | -1·49 (-4·09- 1·09) | 0·26 | Ghanaians in Amsterdam | -4·39 (-6·54- -2·24) | <0·001 |
| *Model 2* | *Change (mmHg) (95%CI)* | *p-value* | ***Model 2*** | *Change (mmHg) (95%CI)* | *p-value* |
| Rural Ghana | Ref. |  | Rural Ghana | Ref. |  |
| Urban Ghana | 1·78 (-1·22- 4·77) | 0·25 | Urban Ghana | -3·48 (-5·69- -1·27) | 0·002 |
| Ghanaians in Amsterdam | -1·49 (-4·22- 1·25) | 0·29 | Ghanaians in Amsterdam | -4·10 (-6·42- -1·78) | <0·001 |
| *Model 3* | *Change (mmHg) (95%CI)* | *p-value* | ***Model 3*** | *Change (mmHg) (95%CI)* | *p-value* |
| Rural Ghana | Ref. |  | Rural Ghana | Ref. |  |
| Urban Ghana | 0·44 (-2·74- 3·62) | 0·78 | Urban Ghana | -4·15 (-6·59- -1·81) | 0·001 |
| Ghanaians in Amsterdam | -2·61 (-5·62- 0·40) | 0·09 | Ghanaians in Amsterdam | -4·77 (-7·26- -2·29) | <0·001 |

*CI, confidence interval.*

*Model 1 is adjusted for age at baseline, follow-up time, and baseline systolic blood pressure;*

*Model 2 is adjusted for model 1 plus education;*

*Model 3 is adjusted for model 2 plus body mass index, estimated glomerular filtration rate, and diabetes mellitus.*

**Supplementary Table 4 – Change in systolic blood pressure in Ghanaians in urban Ghana and Amsterdam relative to rural Ghana, in men and women, excluding those on blood pressure lowering medication**

| Men |  |  | Women |  |  |
| --- | --- | --- | --- | --- | --- |
| *Model 1* | Change (mmHg) (95%CI) | p-value | ***Model 1*** | Change (mmHg) (95%CI) | p-value |
| Rural Ghana | Ref. |  | Rural Ghana | Ref. |  |
| Urban Ghana | 1·66 (-1·15-4·49) | 0·25 | Urban Ghana | -2·83 (-4·93--0·73) | 0·008 |
| Ghanaians in Amsterdam | -1·02 (-3·5-1·5) | 0·42 | Ghanaians in Amsterdam | -5·12 (-7·17--3·06) | <0·001 |
| *Model 2* |  |  | ***Model 2*** |  |  |
| Rural Ghana | Ref. |  | Rural Ghana | Ref. |  |
| Urban Ghana | 2·47 (-0·47-5·43) | 0·1 | Urban Ghana | -2·89 (-5·05--0·73) | 0·009 |
| Ghanaians in Amsterdam | -0·81 (-3·45-1·82) | 0·54 | Ghanaians in Amsterdam | -4·73 (-6·91--2·55) | <0·001 |
| *Model 3* |  |  | ***Model 3*** |  |  |
| Rural Ghana | Ref. |  | Rural Ghana | Ref. |  |
| Urban Ghana | 1·31 (-1·80-4·42) | 0·41 | Urban Ghana | -3·25 (-5·55--0·96) | 0·006 |
| Ghanaians in Amsterdam | -2·10 (-4·98-0·78) | 0·15 | Ghanaians in Amsterdam | -5·18 (-7·52--2·83) | <0·001 |

*CI, confidence interval.*

*Model 1 is adjusted for age at baseline, follow-up time, baseline systolic blood pressure, use of blood pressure lowering medication at baseline, blood pressure lowering medication at follow-up;*

*Model 2 is adjusted for model 1 plus education;*

*Model 3 is adjusted for model 2 plus body mass index, estimated glomerular filtration rate, and diabetes mellitus.*

**Supplementary Table 5 – Incident rate ratio for hypertension in Ghanaians in urban Ghana and Amsterdam relative to rural Ghana, in men and women, with adjustment for additional covariates**

| Men |  |  | Women |  |  |
| --- | --- | --- | --- | --- | --- |
| *Model 2* | *IRR (95% CI)* | *p-value* | ***Model 2*** | *IRR (95% CI)* | *p-value* |
| Rural Ghana | Ref. |  | Rural Ghana | Ref. |  |
| Urban Ghana | 1·12 (0·89-1·41) | 0·35 | Urban Ghana | 0·98 (0·84-1·17) | 0·82 |
| Ghanaians in Amsterdam | 1·04 (0·83-1·29) | 0·74 | Ghanaians in Amsterdam | 0·87 (0·82-1·14) | 0·75 |
| *Model 2b* | *IRR (95% CI)* | *p-value* | ***Model 2b*** | *IRR (95% CI)* | *p-value* |
| Rural Ghana | Ref. |  | Rural Ghana | Ref. |  |
| Urban Ghana | 1·11 (0·88-1·40) | 0·37 | Urban Ghana | 0·98 (0·84-1·13) | 0·73 |
| Ghanaians in Amsterdam | 1·03 (0·82-1·28) | 0·80 | Ghanaians in Amsterdam | 0·97 (0·82-1·12) | 0·57 |
| *Model 3* | *IRR (95% CI)* | *p-value* | ***Model 3*** | *IRR (95% CI)* | *p-value* |
| Rural Ghana | Ref. |  | Rural Ghana | Ref. |  |
| Urban Ghana | 1·09 (0·86-1·39) | 0·47 | Urban Ghana | 0·98 (0·83-1·15) | 0·82 |
| Ghanaians in Amsterdam | 1·01 (0·80-1·28) | 0·94 | Ghanaians in Amsterdam | 0·98 (0·82-1·16) | 0·80 |
| *Model 3b* | *IRR (95% CI)* | *p-value* | ***Model 3b*** | *IRR (95% CI)* | *p-value* |
| Rural Ghana | Ref. |  | Rural Ghana | Ref. |  |
| Urban Ghana | 1·09 (0·86-1·39) | 0·46 | Urban Ghana | 0·99 (0·84-1·16) | 0·90 |
| Ghanaians in Amsterdam | 1·01 (0·80-1·28) | 0·93 | Ghanaians in Amsterdam | 0·99 (0·83-1·18) | 0·93 |
| *Model 4* | *IRR (95% CI)* | *p-value* | ***Model 4*** | *IRR (95% CI)* | *p-value* |
| Rural Ghana | Ref. |  | Rural Ghana | Ref. |  |
| Urban Ghana | 1·09 (0·85-1·39) | 0·49 | Urban Ghana | 0·98 (0·83-1·16) | 0·81 |
| Ghanaians in Amsterdam | 1·00 (0·78-1·29) | 0·98 | Ghanaians in Amsterdam | 0·97 (0·81-2·26) | 0·74 |
| *Model 5* | *IRR (95% CI)* | *p-value* | ***Model 5*** | *IRR (95% CI)* | *p-value* |
| Rural Ghana | Ref. |  | Rural Ghana | Ref. |  |
| Urban Ghana | 1·06 (0·76-1·47) | 0·74 | Urban Ghana | 0·99 (0·81-1·21) | 0·92 |
| Ghanaians in Amsterdam | 1·04 (0·69-1·55) | 0·84 | Ghanaians in Amsterdam | 0·94 (0·73-1·22) | 0·67 |
| *Model 6* | *IRR (95% CI)* | *p-value* | ***Model 6*** | *IRR (95% CI)* | *p-value* |
| Rural Ghana | Ref. |  | Rural Ghana | Ref. |  |
| Urban Ghana | 1·06 (0·76-1·48) | 0·75 | Urban Ghana | 0·99 (0·81-1·22) | 0·96 |
| Ghanaians in Amsterdam | 1·01 (0·65-1·52) | 0·96 | Ghanaians in Amsterdam | 0·94 (0·72-1·21) | 0·62 |

*CI, confidence interval.*

*Model 2 adjusted for age at baseline, follow-up time, baseline systolic and diastolic blood pressure, level of education;*

*Model 2b adjusted for model 2 and change in BMI between baseline and follow-up;*

*Model 3 adjusted for model 2 and baseline BMI, eGFR and diabetes;*

*Model 3b adjusted model 3 and waist-to-hip ratio;*

*Model 4 adjusted for model 3 and family history of hypertension*

*Model 5 adjusted for model 4 and physical activity, physical activity, total energy intake, smoking, alcohol intake and sodium intake;*

*Model 6 adjusted for model 5 plus psychosocial stress and depression*

**Supplementary Table 6 – Incident rate ratio for hypertension in Ghanaians in Amsterdam relative to Dutch, in men and women, with adjustment for additional covariates**

| Men |  |  | Women |  |  |
| --- | --- | --- | --- | --- | --- |
| *Model 2* | *IRR (95% CI)* | *p-value* | ***Model 2*** | *IRR (95% CI)* | *p-value* |
| Dutch | Ref. |  | Dutch | Ref. |  |
| Ghanaians in Amsterdam | 1·02 (0·83-1·25) | 0·84 | Ghanaians in Amsterdam | 1·12 (0·94-1·34) | 0·21 |
| *Model 2b* | *IRR (95% CI)* | *p-value* | ***Model 2b*** | *IRR (95% CI)* | *p-value* |
| Dutch | Ref. |  | Dutch | Ref. |  |
| Ghanaians in Amsterdam | 1·06 (0·89-1·26) | 0·51 | Ghanaians in Amsterdam | 1·12 (0·98-1·29) | 0·10 |
| *Model 3* | *IRR (95% CI)* | *p-value* | ***Model 3*** | *IRR (95% CI)* | *p-value* |
| Dutch | Ref. |  | Dutch | *Ref.* |  |
| Ghanaians in Amsterdam | 1·03 (0·82-1·28) | 0·80 | Ghanaians in Amsterdam | 1·12 (0·92-1·36) | 0·24 |
| *Model 3b* | *IRR (95% CI)* | *p-value* | ***Model 3b*** | *IRR (95% CI)* | *p-value* |
| Dutch | Ref. |  | Dutch | *Ref.* |  |
| Ghanaians in Amsterdam | 1·04 (0·83-1·30) | 0·73 | Ghanaians in Amsterdam | 1·12 (0·92-1·36) | 0·24 |
| *Model 4* | *IRR (95% CI)* | *p-value* | ***Model 4*** | *IRR (95% CI)* | *p-value* |
| Dutch | Ref. |  | Dutch | Ref. |  |
| Ghanaians in Amsterdam | 1·05(0·81-1·35) | 0·71 | Ghanaians in Amsterdam | 1·11 (0·88-1·39) | 0·38 |

*CI, confidence interval.*

*Model 2 adjusted for age at baseline, follow-up time, baseline systolic and diastolic blood pressure, level of education;*

*Model 2b adjusted for model 2 and change in BMI between baseline and follow-up;*

*Model 3 adjusted for model 2 and baseline BMI, eGFR, diabetes mellitus;*

*Model 3b adjusted for model 3 and waist-to-hip ratio;*

*Model 4 adjusted for model 3 and family history of hypertension.*

**Supplementary Table 7 – Incident rate ratio for hypertension and change in systolic blood pressure after multiple imputation of missing covariates, in Ghanaians in urban Ghana and Amsterdam relative to rural Ghana, and in Ghanaians in Amsterdam relative to Dutch**

| Incident hypertension | |  |  | Delta systolic blood pressure | | |  |  |
| --- | --- | --- | --- | --- | --- | --- | --- | --- |
| Men |  | IRR (95% CI) | p-value | **Men** |  | Change (mmHg) (95% CI) | | p-value |
| *Rural Ghana* |  | Reference |  | *Rural Ghana* |  | Reference | |  |
| *Urban Ghana* | Model 1 | 1·07 (0·86-1·34) | 0·53 | *Urban Ghana* | Model 1 | 2·75 (-0·85-6·35) | | 0·13 |
|  | Model 2 | 1·09 (0·87-1·37) | 0·45 |  | Model 2 | 3·03 (-0·71-6·76) | | 0·11 |
|  | Model 3 | 1·06 (0·83-1·36) | 0·63 |  | Model 3 | 0·95 (-3·04-4·94) | | 0·64 |
| *Ghanaians in Amsterdam* | Model 1 | 1·01 (0·82-1·25) | 0·92 | *Ghanaians in Amsterdam* | Model 1 | -0·61 (-4·03-2·8) | | 0·72 |
|  | Model 2 | 1·03 (0·82-1·28) | 0·80 |  | Model 2 | -0·34 (-3·92-3·25) | | 0·85 |
|  | Model 3 | 0·99 (0·77-1·28) | 0·95 |  | Model 3 | -2·75 (-6·81-1·31) | | 0·18 |
|  |  | IRR (95% CI) | p-value |  |  | Change (mmHg) (95% CI) | | p-value |
| *Dutch* |  | Reference |  | *Dutch* |  | Reference | |  |
| *Ghanaians in Amsterdam* | Model 1 | 1·09 (0·92-1·28) | 0·30 | *Ghanaians in Amsterdam* | Model 1 | 3·74 (1·75-5·74) | | 0·00 |
|  | Model 2 | 1·03 (0·84-1·26) | 0·78 |  | Model 2 | 1·6 (-0·83-4·03) | | 0·20 |
|  | Model 3 | 1·04 (0·84-1·28) | 0·73 |  | Model 3 | 1·48 (-1·1-4·05) | | 0·26 |
| Women |  | IRR (95% CI) | p-value | **Women** |  | Change (mmHg) (95% CI) | | p-value |
| *Rural Ghana* |  | Reference |  | *Rural Ghana* |  | Reference | |  |
| *Urban Ghana* | Model 1 | 0·98 (0·84-1·13) | 0·75 | *Urban Ghana* | Model 1 | -1·49 (-4·09-1·11) | | 0·26 |
|  | Model 2 | 0·98 (0·84-1·14) | 0·79 |  | Model 2 | -1·59 (-4·23-1·05) | | 0·24 |
|  | Model 3 | 0·97 (0·83-1·15) | 0·76 |  | Model 3 | -1·9 (-4·75-0·96) | | 0·19 |
| *Ghanaians in Amsterdam* | Model 1 | 0·96 (0·83-1·12) | 0·64 | *Ghanaians in Amsterdam* | Model 1 | -4·42 (-7·11--1·73) | | 0·00 |
|  | Model 2 | 0·97 (0·83-1·14) | 0·73 |  | Model 2 | -4·25 (-7·1--1·41) | | 0·00 |
|  | Model 3 | 0·97 (0·81-1·15) | 0·69 |  | Model 3 | -4·56 (-7·6--1·51) | | 0·00 |
|  |  | IRR (95% CI) | p-value |  |  | Change (mmHg) (95% CI) | | p-value |
| *Dutch* |  | Reference |  | *Dutch* |  | Reference | |  |
| *Ghanaians in Amsterdam* | Model 1 | 1·14 (1-1·3) | 0·04 | *Ghanaians in Amsterdam* | Model 1 | 7·29 (5·65-8·93) | | 0·00 |
|  | Model 2 | 1·12 (0·94-1·33) | 0·20 |  | Model 2 | 6·22 (4·06-8·37) | | 0·00 |
|  | Model 3 | 1·12 (0·93-1·34) | 0·24 |  | Model 3 | 5·20 (2·89-7·50) | | 0·00 |

*IRR, incidence rate ratio; CI, confidence interval.*

*Model 1 adjusted for age at baseline, follow-up time, and baseline systolic blood pressure;*

*Model 2 adjusted for is adjusted for age, follow-up time, baseline SBP (plus 10 mmHg for those on blood pressure lowering medication), level of education;*

*Model 3 adjusted for model 2 plus BMI, eGFR, diabetes mellitus at baseline*


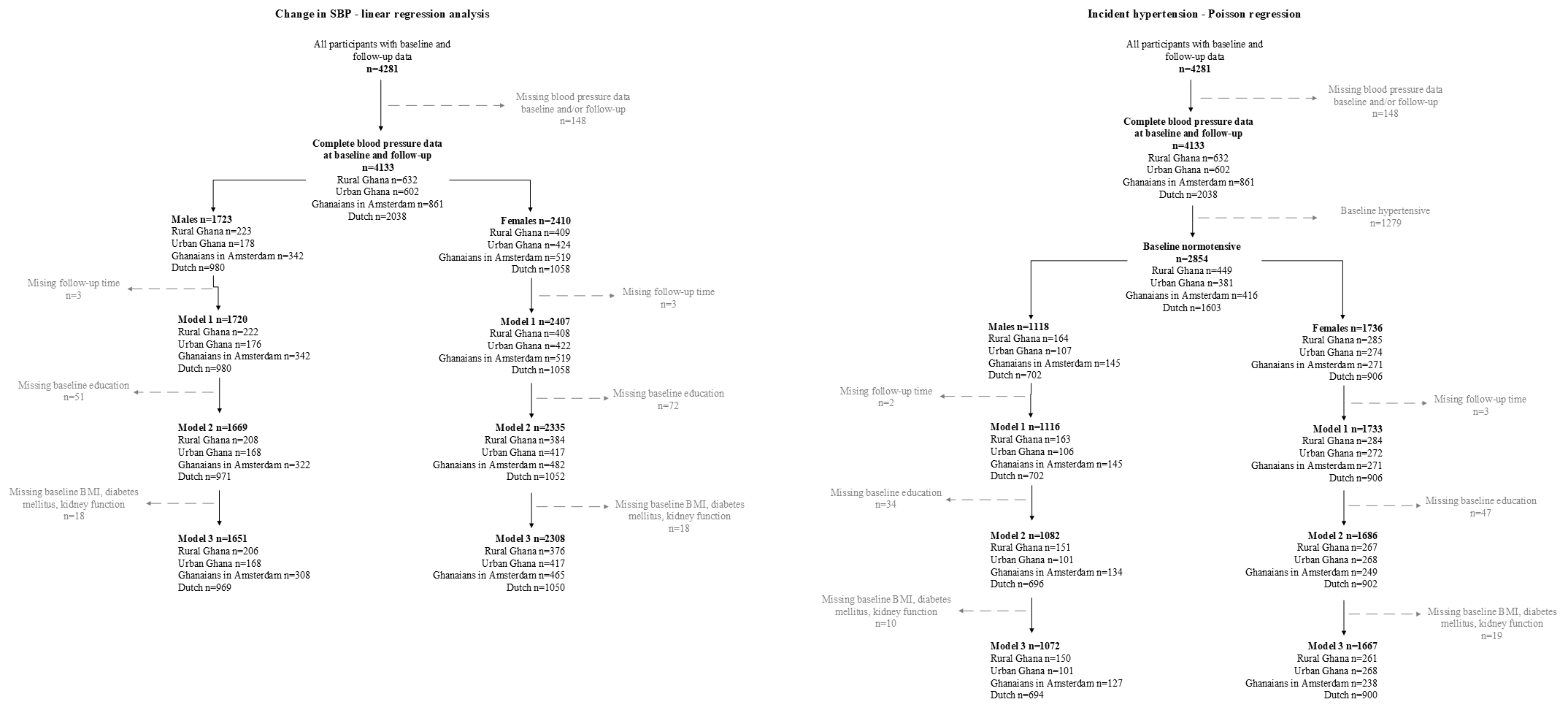
**Supplementary Figure 1** **– Flowchart of participants included in the analyses for change in systolic blood pressure (A) and incident hypertension (B)**

**b**

**a**

*Model 1 was adjusted for age at baseline and follow-up time; model 2 was adjusted for age, follow-up time and education; model 3 was adjusted for model 2 plus body mass index, estimated glomerular filtration rate and diabetes mellitus. BMI, body mass index; eGFR, estimated glomerular filtration rate, SBP, systolic blood pressure.*

**Supplementary Figure 2 – Incident rate ratio for in Ghanaians in urban Ghana and Amsterdam relative to rural Ghana, in men (A) and women (B) amongst those without hypertension at baseline**

**a**

**b**


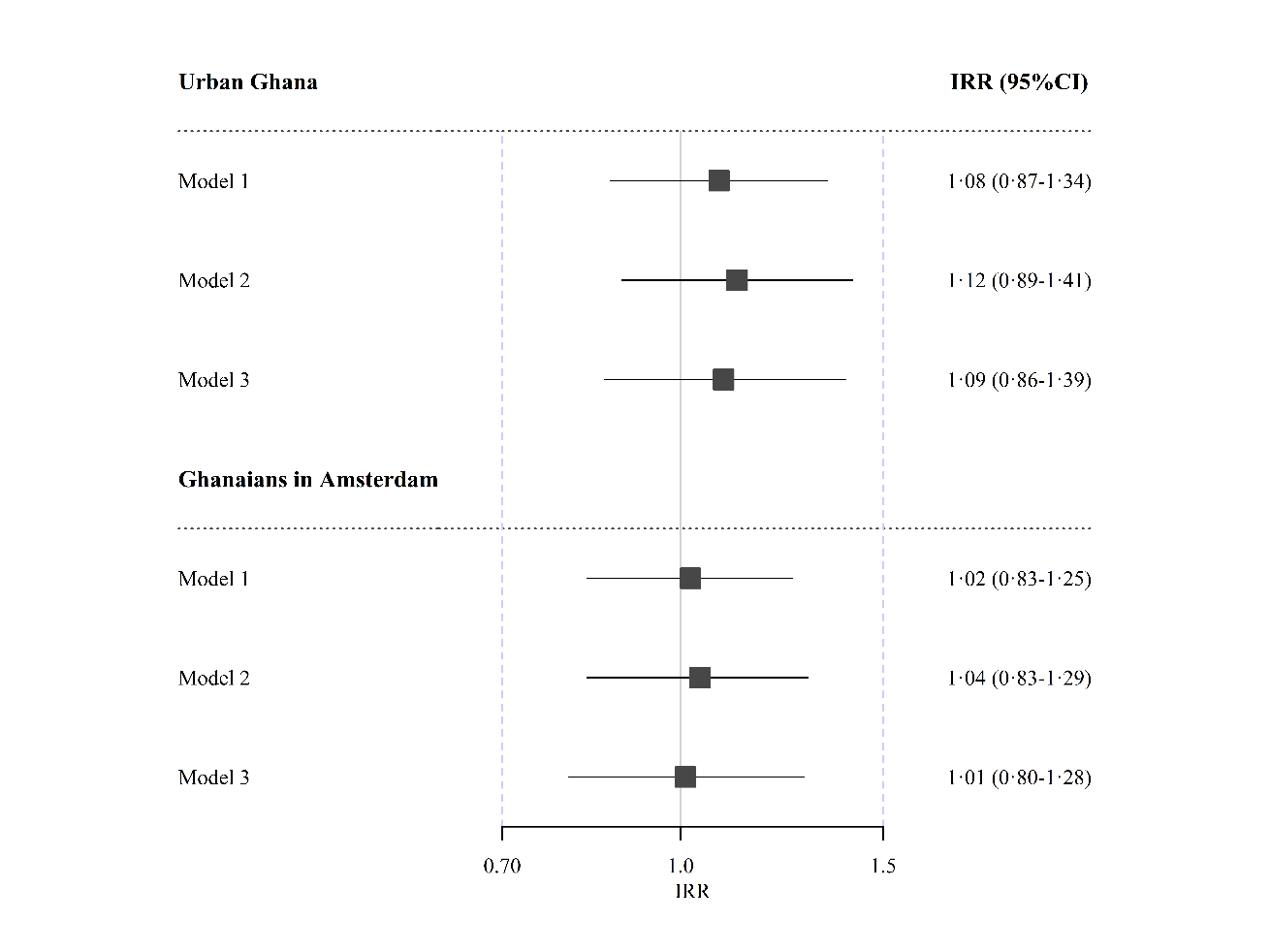

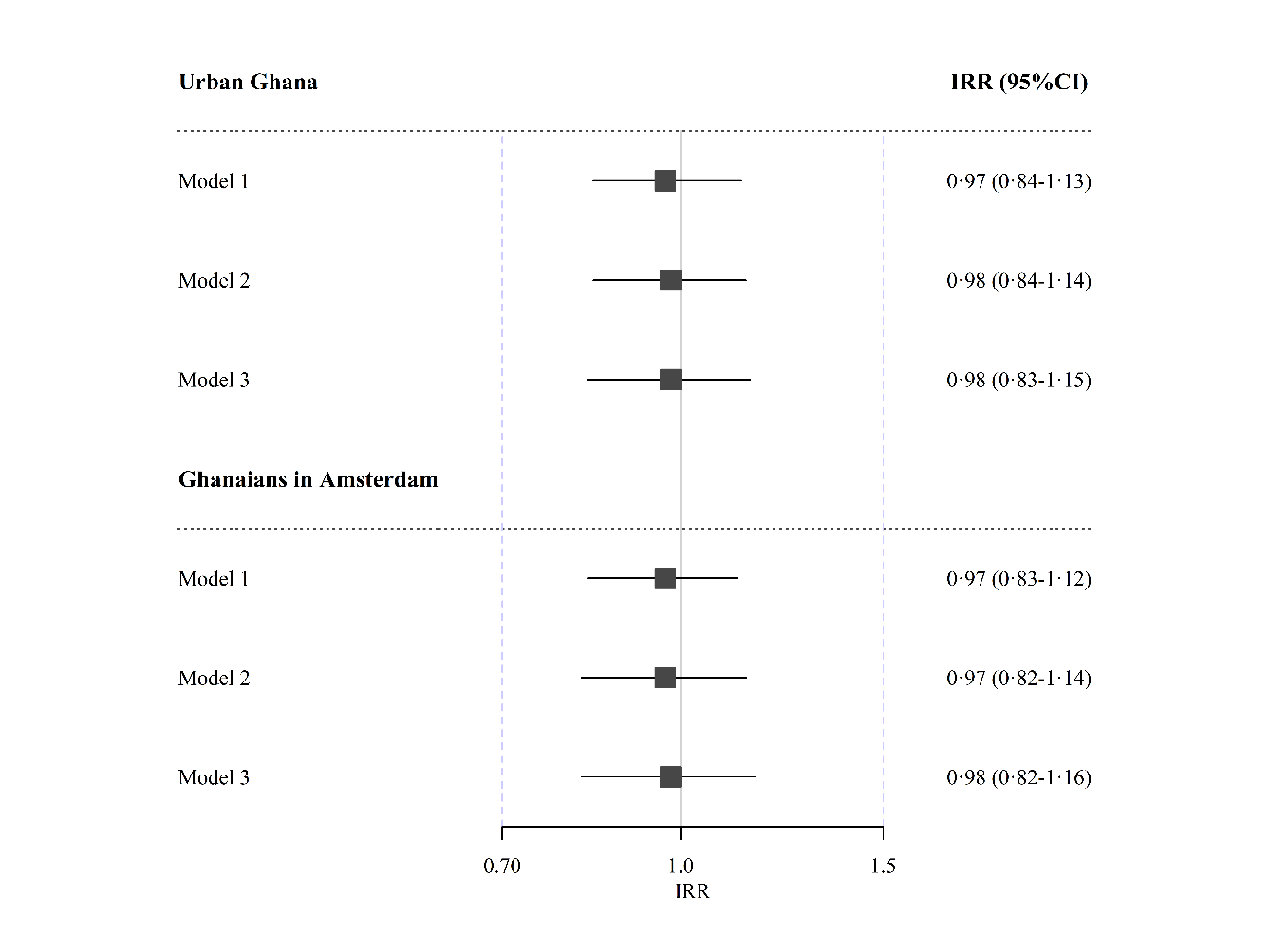


*Model 1 is adjusted for age at baseline, follow-up time, baseline systolic and diastolic blood pressure; model 2 is adjusted for variables in model 1 plus education; model 3 is adjusted for model 2 plus body mass index, estimated glomerular filtration rate, and diabetes mellitus. IRR, incidence rate ratio; CI, confidence interval.*

**Supplementary Figure 3 – Incident rate ratio for hypertension in Ghanaians in Amsterdam relative to Dutch, in men (A) and women (B) amongst those without hypertension at baseline**





*Model 1 is adjusted for age at baseline, follow-up time, baseline systolic and diastolic blood pressure; model 2 is adjusted for variables in model 1 plus education; model 3 is adjusted for model 2 plus body mass index, estimated glomerular filtration rate, and diabetes mellitus. IRR, incidence rate ratio; CI, confidence interval*
